# RarERN Path: a methodology towards the optimisation of patients’ care pathways in rare and complex diseases developed within the European Reference Networks

**DOI:** 10.1186/s13023-020-01631-1

**Published:** 2020-12-14

**Authors:** Talarico Rosaria, Cannizzo Sara, Lorenzoni Valentina, Marinello Diana, Palla Ilaria, Pirri Salvatore, Ticciati Simone, Trieste Leopoldo, Triulzi Isotta, Terol Enrique, Bucher Anna, Turchetti Giuseppe

**Affiliations:** 1 Rheumatology Unit, Azienda Ospedaliero Universitaria Pisana, 56126 Pisa, Italy; 2 Institute of Management, Scuola Superiore Sant’Anna, Piazza Martiri della Libertà, 33, 56127 Pisa, Italy; 3 DG Health and Food Safety, 1000 Brussels, Belgium

**Keywords:** Rare diseases, Patients’ care pathways, European Reference Networks, Complex diseases, Patients involvement, Method, Organisation of care, Health economics, Health technology assessment.

## Abstract

**Background:**

In 2017, the European Commission has launched the European Reference Networks (ERNs), virtual networks involving healthcare providers across Europe. The aim of the ERNs is to tackle complex and rare diseases and conditions that require highly specialized treatment and a concentration of knowledge and resources. The ERN on rare and complex connective tissue and musculoskeletal diseases (ERN ReCONNET) is one of the 24 ERNs approved that aims to improve the management of Rare and Complex Connective Tissue and Musculoskeletal Diseases.

**Objective:**

The RarERN Path methodology aims to create a single reference organisational model for patients’ care pathways which, if applied in different contexts, helps to ensure an improved, cost-effective and patient-centred equal care to rare and complex diseases.

**Methods:**

Starting from existing standard methods for the creation and elaboration of patients’ care pathways, a specific methodology was created in order to take advantage of the distinctive and peculiar characteristics of the ERNs. Specifically, the development of the RarERN Path methodology involved different stakeholders: health economists, clinicians and researchers expert in rare and complex diseases, communication experts, experts in patients’ involvement and narrative medicine and policy-makers.

**Results:**

The RarERN Path methodology foresees six consecutive phases, each with different and specific aims. Specifically, the six phases are represented by: Phase 1—mapping of existing patients’ care pathways and patients’ stories; Phase 2—design of an optimised common patients’ care pathway; Phase 3—consensus on an optimised common patients’ care pathway; Phase 4—key performance indicators definition; Phase 5—refinement; Phase 6—pilot phase (optional).

**Conclusion:**

The application of RarERN Path to the different disease-specific and geographical contexts would help to ensure an improved, cost-effective and patient-centred equal care to rare and complex diseases across Europe as well as a possible tangible action towards the integration of ERNs into the different European healthcare systems.

## Background

More than 30 million persons in Europe are affected by rare diseases (RDs). Due to their low prevalence, knowledge and expertise on RDs can be limited and access to diagnosis, high-quality care and treatments are often unequal throughout Europe. In order to address the current challenges, the European Commission has launched an important initiative that, starting from March 2017, has established the European Reference Networks (ERNs), virtual networks involving healthcare providers (HCPs) across Europe [1]. The aim of the ERNs is to tackle complex and rare diseases and conditions (RCD) that require highly specialized treatment and a concentration of knowledge and resources. It is well known that no country alone has the knowledge and capacity to treat all RCD and for this very reason, ERNs offer the potential to give patients and clinicians across the EU access to the best expertise and timely exchange of life-saving competence, making knowledge travel more rather than patients [2]. Twenty-four ERNs are currently working on different thematic areas, such as rare cancers, neuromuscular and metabolic disorders [3]. The ERN ReCONNET [4] is one of the 24 ERNs approved that aims to improve the management of Rare and Complex Connective Tissue and Musculoskeletal Diseases (rCTDs) across the EU. In addition, the ERN ReCONNET involves and engages with patient organizations thanks to the creation of the ERN ReCONNET European Patients Advocacy Group (ePAG) [5].

In order to implement the mission of the ERNs and to ensure their sustainability, the ERNs are expected to create a precise and stable link with the different healthcare systems of the Member States. This was clearly defined in the *Statement of the ERN Board of Member States (BoMS) on Integration of the ERNs to the healthcare systems of Member States* [6], in which the BoMS outlines tangible actions towards the integration of the ERNs into the Member States, prioritizing and encouraging specific actions aimed at planning and implementing the integration process. Among the specific actions mentioned in the Statement, the BoMs encourages the creation of appropriate (clear and well-defined) patients’ care pathways (PCP) in order to improve the care and the management of patients with RCD.

The European Pathway Association defines the PCP as a “complex intervention for the mutual decision making and organisation of care processes for a well-defined group of patients during a well-defined period.” [7]. Therefore, it seems clear that the main goal of a PCP is to enhance the quality of care by providing an integrated tool for the treatment of several complex diseases; this is particularly crucial in the field of RCD.

Taking into consideration the mission of improving the care of RDs patients in Europe and considering the multi-stakeholder involvement, ERNs might represent the most appropriate setting for the creation of organisational reference models for PCP across Europe. For this reason, in the framework of the collaboration between the ERN ReCONNET Coordination Team and the group of Health Economics of the Institute of Management of the Scuola Superiore Sant’Anna, an extensive effort has been made towards the creation of an organisational reference model for PCP in RCD across the different Member States. In order to develop the reference model, a specific methodology was created to enable the design of the PCP based on a deep sharing of expertise on high-quality care and characterized by a strong patient-centred approach. The development of this specific methodology started from the need to implement the existing approaches already in use for the assessment of PCP.

An ad hoc methodology was in fact needed to address the specificity and the innovative asset provided by the ERNs and their unique environment represented by a multi-national and multi-stakeholder collaborative framework. The existing methodologies already in use to create and assess PCP were in fact implemented, adjusted and largely upgraded to the larger context of the ERNs, both in terms of countries and healthcare providers’ involvement as well as to the peculiar characteristics of RCD and the need to enable the adaptation of the PCP in the different healthcare systems.

This work is aimed at reporting on RarERN Path, a methodology specifically designed to develop an organisational reference model for PCP in RCD, that can be adapted in a flexible way to different disease-specific and geographical contexts. Specifically, RarERN Path, represents, in this vision, a tangible example of the application of the “Share. Care. Cure” fundamental principle of ERNs.

PCP represent health management tools that indicate the sequence of the procedures that need to be carried out on the basis of the current scientific knowledge and on the available organizational, professional and technological resources [8–13]. The approach used to assess and analyse PCP is related to the general concept of process management that considers the path of the patient from an organisational point of view. In particular, the process management aims to ensure the effectiveness, the efficiency, and the management of the care while improving the quality of the patient’s experience and care.

It is well known that the concept of PCP does not overlap with Clinical Practice Guidelines (CPGs), but can rather represent a tangible organisational strategy to implement and apply CPGs not only at national level, but also in the single healthcare provider’s environment. The CPGs are defined as “statements that include recommendations intended to optimize patient care that are informed by a systematic review of evidence and an assessment of the benefits and harms of alternative care options” [14]; therefore, it is clear that defining and improving PCP may surely contribute to a more efficient and sustainable application of the CPGs, especially in the case of health contexts where less expertise is available and the resources for care are limited.

This is particularly crucial in the field of RCD, where the knowledge is often scattered and access to care and treatment can be heterogeneous. In this scenario, it appears clear that PCP can have an evident role in accomplishing ERNs’ mission to facilitate an equitable access to timely diagnosis and delivery of high quality, accessible and cost-effective healthcare for all patients with RCD. PCP are in fact considered crucial in providing patient-centered care and in the promotion of an efficient use of resources [13].

For this reason, ERNs can provide important added value in the implementation of PCP especially considering their multi-stakeholders and multi-countries nature, taking advantage of the high level of expertise that characterizes the Networks and building on the well-established patient-centered approach that defines the ERNs themselves.

Following these considerations and taking into account the work already in place in the ERN ReCONNET on organisational and economic aspects of RCD [15], it appeared clear that one of the needs to be prioritised was represented by the development of a specific methodology aimed at defining organisational PCP models across Europe.

## Objective

The object of this paper is to provide a careful description of the RarERN Path methodology, that aims to create a single reference organisational model for PCP which, if applied in different contexts, helps to ensure an improved, cost-effective and patient-centred equal care to RCD.

## Methods

Starting from existing standard methods for the creation and elaboration of PCP, a specific methodology was created in order to take advantage of the distinctive and peculiar characteristics of the ERNs; the integration of the perspectives of large communities of patients, expert clinicians, health economists and healthcare providers from different EU countries surely represents a real innovative approach towards the creation of organisational models of PCP for RCD that are integrated, flexible and adaptable to the different health systems in Europe. The development of the RarERN Path methodology involved the following stakeholders: health economists, clinicians and researchers expert in RCD, communication experts, experts in patients’ involvement and narrative medicine and policy-makers. Specifically, the stakeholders involved in the development of this methodology convened on a regular basis to integrate the existing methods aimed at mapping clinical pathways with the experience of the Coordination of a European Network as well as with the expertise of the different stakeholders involved. The design of each specific phase was performed by means of dedicated consensus meetings that involved all the stakeholders mentioned. After the final consensus of the whole methodology, an internal validation was performed by simulating the application the RarERN Path methodology in a specific rare disease with the support of all the stakeholders involved in the design.

## Results

### RarERN Path

**Fig. 1:**
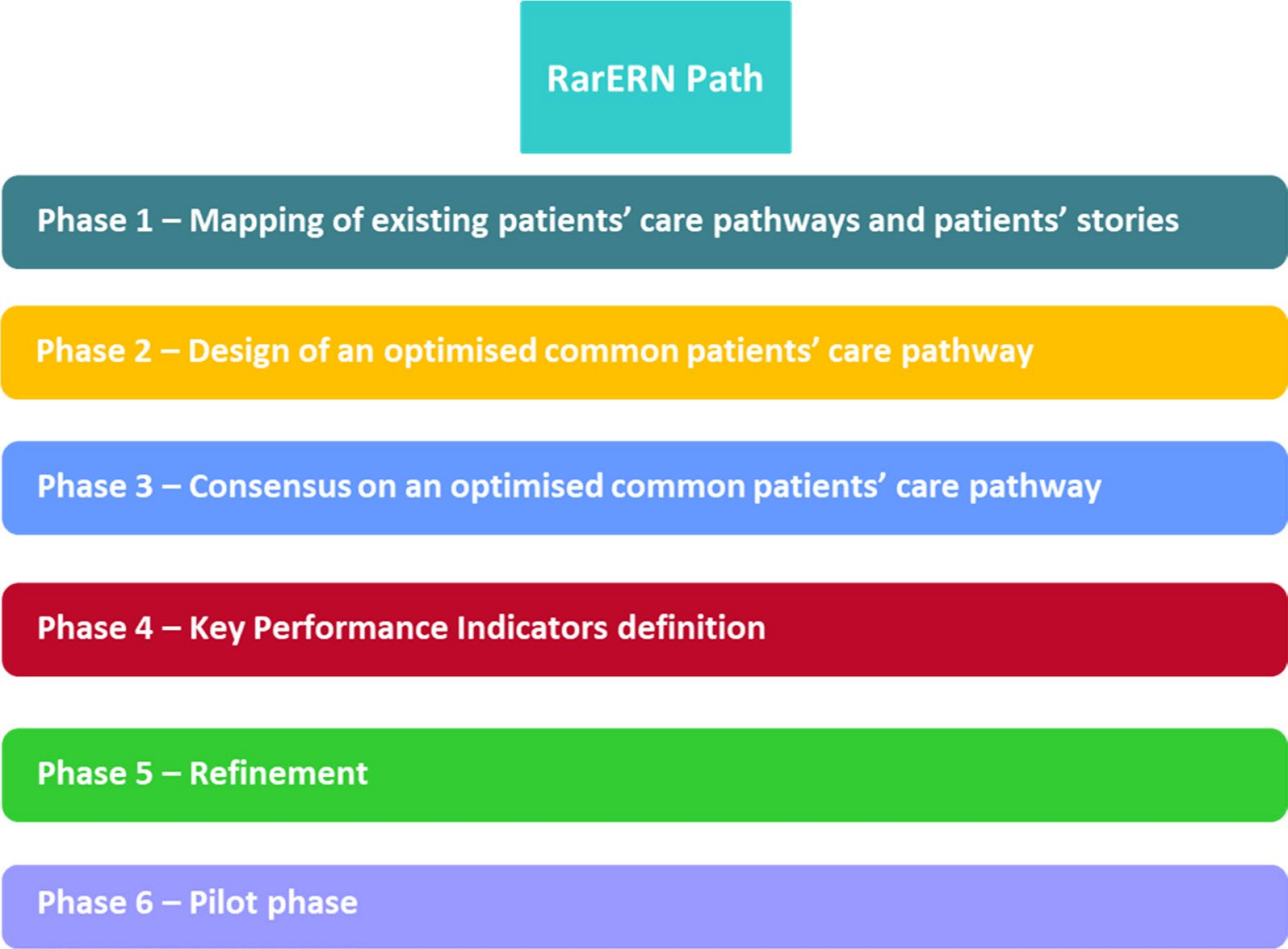
RarERN Path phases

The RarERN Path approach foresees six consecutive phases (see Fig. 1), each with different and specific aims (Table 1).

**Table 1:** RarERN Phases and expected outputs

Phase	Aim	Output
Phase 1—mapping of existing patients’ care pathways and patients’ stories	To get the picture of the current practice in rCTDs care organizations across the different ERN ReCONNET centres and collect patients’ stories and perspectives on their experience with their care and their disease	(1a) Flowchart that graphically represents the different phases of the care pathway followed in each centre (1b) Co-design of a survey for the collection of patients’ stories (possibly in different languages)
Phase 2—design of an optimised patients’ care pathway	Optimization of the current care provided to patients in a common patients’ care pathway and elaboration of patients’ stories	(2a) Drafting of the optimised patients’ care pathway Flowchart taking into account the patients’ care pathways followed in the HCPs for diagnosis, treatment and monitoring (2b) Integration of the list of needs and priorities related to care and care pathway extrapolated from the patients’ stories into the common patients’ care pathway Flowchart
Phase 3—consensus on an optimised patients’ care pathway	To reach a consensus among stakeholders on an optimised reference organisational model on the patients’ care pathway to be followed for the specific disease	Optimised reference organisational model on the patients’ care pathway to be followed for the specific disease
Phase 4—KPI definition	Co-design of Key Performance Indicators (KPI) needed to assess the performance of the organisational procedures, their impact on the disease outcome and the economic and organisational sustainability for the healthcare providers	Detailed list of the Key Performance Indicators (KPI)
Phase 5—refinement	Development of the final version of the optimised common care pathway model, of related KPIs, and of instructions for its application in specific healthcare contexts	Inclusion of additional KPIs in the optimised common reference organisational model on the patients’ care pathway to be followed for the specific disease. Dissemination of the optimised common reference organisational model across the different stakeholders
Phase 6—pilot phase (optional)	To assess the applicability and adaptability of the optimised common patients’ care pathway in specific healthcare providers using related KPIs	Application of the organisational model and collection of the KPIs. Identification of eventual barriers to the application of the organisational flow in specific contexts

# Phase 1—mapping of existing patients’ care pathways and patients’ stories.

# Phase 2—design of an optimised common patients’ care pathway.

# Phase 3—consensus on an optimised common patients’ care pathway.

# Phase 4—key performance indicators (KPI) definition.

# Phase 5—refinement.

# Phase 6—pilot phase.

### # Phase 1: mapping of existing patients’ care pathways and patients’ stories

*Aim *To get the picture of the current practice in rCTDs care organizations across the different ERN ReCONNET centres and collect patients’ stories and perspectives on their experience with their care and their disease.

#### Phase 1a: collection of existing patients’ care pathways

Phase 1a is dedicated to the mapping of existing PCP followed in all the HCPs of the Network by means of an ad hoc questionnaire; the questionnaire focuses on the organizational analysis of the PCP that patients with rCTDs follow from the referral to the follow-up in each centre involved in the Network for each disease included in ERN ReCONNET. The results of the questionnaire of each centre should then be transferred into a Flowchart that graphically represents the different phases of the PCP followed by patients in the centre (diagnosis, treatment and monitoring). The Flowchart needs to be validated by the HCP representative, who should carefully review the Flowchart and confirm whether the pathway currently in place in the centre was correctly represented in the Flowchart. The validation process can take place either via email or via web-conference/face to face meeting. In this phase, the HCP Representative also has the possibility to send informative material regarding the PCP used in the centre in order to collect useful additional details on the care offered in the centre.

Expected output: Flowchart that graphically represents the different phases of the patients’ care pathway followed in each centre.

#### Phase 1b: collection of patients’ stories

In order to collect the views and perspectives of patients, a survey based on the principles of narrative medicine is co-designed in English with patients affected by the disease.

The Narrative Medicine is a medical approach developed in the United States in the late Nineties by Rita Charon, Professor of Clinical Medicine and Director of the Program in Narrative Medicine at the Columbia University College of Physicians and Surgeons. This approach is aimed at integrating clinical practice with the stories of illness of patients, enabling healthcare professionals and other actors to understand the perspectives of patients and to address their needs and concerns more effectively. Rita Charon considers narrative medicine “as a new frame for health care, offering the hope that healthcare systems can become more effective thanks to the recognition and the taking into consideration of patients and their experience of illness [16]. The narrative medicine approach, in fact, is particularly valuable as it allows individual patients to tell their stories of their illness, to express their point of view, perceptions and to narrate their experience regarding their care and their pathway [17]. In this perspective, the organisation of survey is an efficient tool to reach a high number of patients affected by the specific disease, highlighting the importance of how the patient perceives the disease and understanding the level of awareness and the experience lived by patients.

The survey consists of:

- an introduction aimed at explaining the scope of the survey and mentioning the eventual further use of the stories,
- a demographic series of questions to have a profile of the responders,
- a free-text space dedicated to write the stories (3600–5000 characters) with a set of questions to support and inspire the patient while telling the story.

Once the survey is finalised, it should then be translated into different EU languages, possibly involving patients and patients’ representatives from the different countries in the validation process. The survey is then launched via the EU Survey Platform [18]—survey platform developed by the European Commission and available for free—across the communities of RCD patients in Europe thanks to the collaboration of the Patients’ Organisations that can play a major role in the dissemination of the survey.

*Expected output* Co-design of a survey for the collection of patients’ stories (possibly in different languages).

### # Phase 2: design of an optimised common patients’ care pathway

*Aim* Optimization of the current care provided to patients in a common patients’ care pathway and elaboration of patients’ stories.

#### Phase 2a: optimization of the current care provided to patients in a common patients’ care pathway

Each validated Flowchart representing the different phases of the PCP in each centre is then merged into a single optimised Flowchart that illustrates a common PCP followed in the HCPs of the ERN for diagnosis, treatment and monitoring. The common PCP Flowchart should include all the common elements and eventual discrepancies identified in the questionnaires, as well as the main challenges and suggestions related to the pathways that were mentioned by clinicians.

**Fig. 2:**
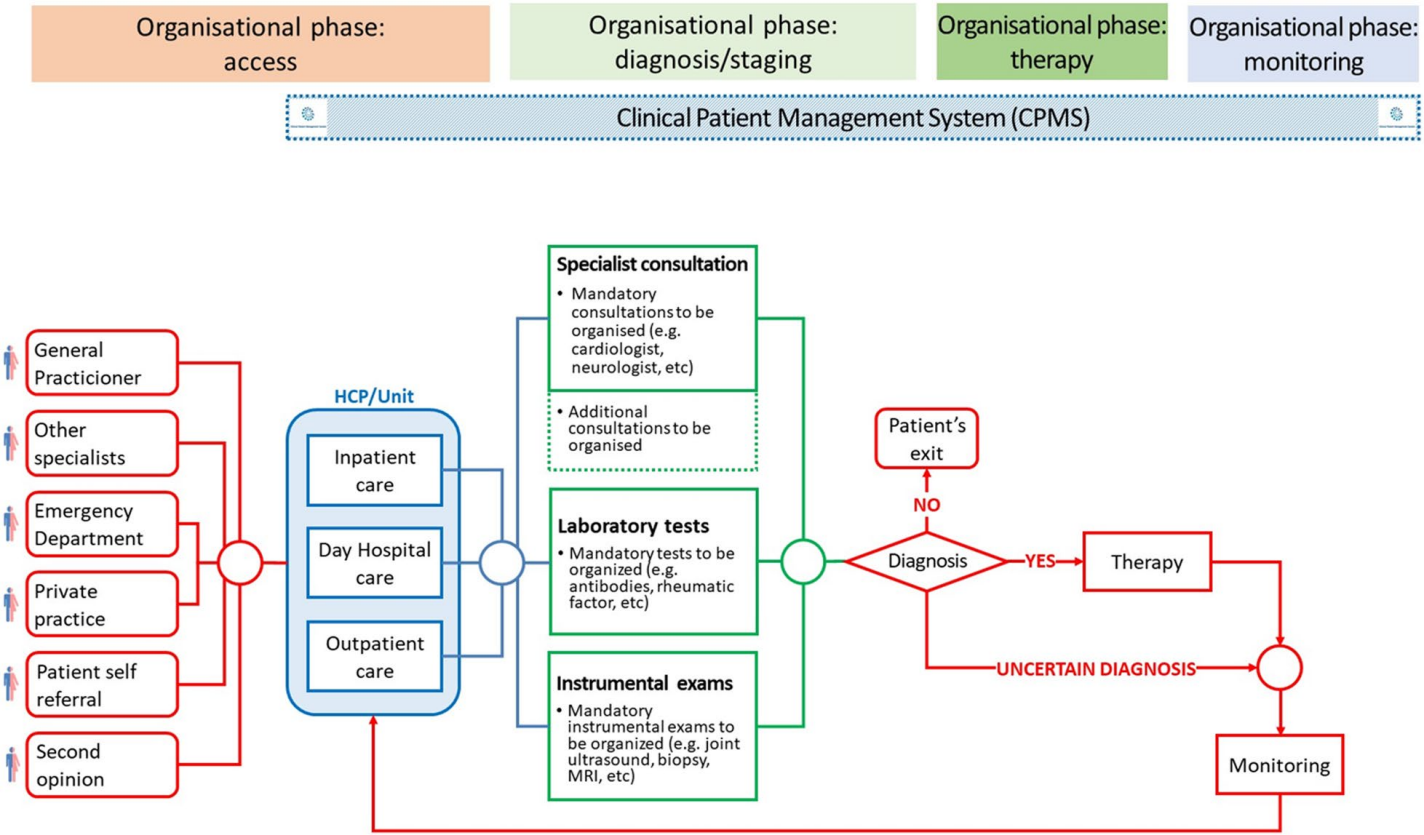
Representation of the organisational phases

*Expected output* Optimised common patients’ care pathway Flowchart taking into account of the patients’ care pathways followed in the HCPs for diagnosis, treatment and monitoring (see example in Fig. 2).

#### Phase 2b: elaboration of patients’ stories

The patients’ stories should be anonymously collected possibly by the team managing the project and reviewed with the patients’ representatives who dealt with the translations in order to identify recurrent topics and specific comments related to patients’ care and pathway. In particular, a list of needs and priorities related to care and care pathway should be identified in the stories for each country by the patients’ representatives with the support of the team managing the project. A final list of the main elements and points presented in the stories is created by merging the feedback collected among the different countries, and it is then validated together with the patients’ representatives. In addition, patients’ stories could also be further explored by analysing the frequency of the words used in order to identify in a word cloud which topics are considered most relevant by patients when talking about their disease and their pathway (Fig. 2). This result can be particularly useful during the discussions to be performed in Phase 3. Other very relevant topics can also be identified with the aim of raising awareness in the clinical and public community on the impact of the disease and to enable a better understanding of the patients’ journey and perspectives.

The list of the main topics described in the patients’ stories should then be integrated into the common PCP Flowchart and with clinicians’ challenges and suggestions.

*Expected output* Integration of the list of needs and priorities related to care and care pathway extrapolated from the patients’ stories into the optimised common patients’ care pathway Flowchart.

### # Phase 3: consensus on an optimised common patients’ care pathway

*Aim* To reach a consensus among stakeholders on an optimisedcommon reference organisational model on the patients’ care pathway to be followed for the specific disease.

In this phase, the different stakeholders analyse together each individual phase (diagnosis, treatment and monitoring) of the optimised common Flowchart of the PCP by means of dedicated working group(s); the working group can be organised in face to face (preferable option) or virtual meetings. The different stakeholders that should participate in the working group include patients and patients’ representatives, caregivers, expert clinicians, health economists and also hospital managers. The working group can use specific tools that enable each participant to provide an opinion on the procedure and on the challenge/topic discussed, for example, by means of coloured cards that can be used to express their point of view raising the appropriate colour (red for challenges, yellow for integration and green for comments). The discussion should also include the challenges, suggestions and eventual discrepancies identified in the clinicians’ questionnaires and in the patients’ stories. During the discussion, any element raised by the participants should be considered and discussed in order to reach the two main goals of the reference organisational model: (1) a common model to be followed by HCPs to ensure an optimised organisational scenario for the management of RCD; (2) to ensure the adaptability and flexibility of the optimised common organisational model in the different HCPs’ settings and in the different healthcare systems.

Each phase must be agreed by the majority of the participants and represented in a graphic flowchart in order to be considered as an ERN optimised common reference organisational model on PCP to be followed for the specific disease (Fig. 2). In order to reach the consensus, the final approval of the final graphic flowchart can take place either via email or via face to face/virtual meetings. A free live discussion is highly suggested to ensure that all stakeholders are able to provide their opinions and interact with each other; however, the consensus can be reached by a formal agreement or a Delphi.

*Expected output* Optimised common reference organisational model on the patients’ care pathway to be followed for the specific disease.

### # Phase 4: KPI definition

*Aim* Co-design of Key Performance Indicators (KPI) needed to assess the performance of the organisational procedures, their impact on the disease outcome and the economic and organisational sustainability for the healthcare providers.

Once the optimised common reference organisational model on PCP to be followed for the specific disease is defined, specific KPIs need to be co-designed with different stakeholders to monitor the following domains in each HCPs:

- Process indicators: to measure how the organisational model may facilitate an effective provision of care (e.g. time from patients’ referral to access to the centre, time to diagnosis, etc.);
- Outcome indicators: to measure how the organisational model can impact on the disease outcome both from the point of view of the clinicians (e.g. how an organised care flow may impact on the disease activity and damage) and of the patients (e.g. satisfaction survey on the care flow and organisation);
- Sustainability indicator: to measure the organisational and economic efficiency.

The KPIs should be co-designed in dedicated working groups (face to face or virtual) and a consensus must be reached among the different stakeholders.

In addition, a set of instructions should be also co-designed in order to be integrated with the optimised common PCP. The instructions might include examples of best practices that can be used to implement a specific procedure, to make patients more aware that they are part of a dedicated pathway, etc. The need to detail pragmatic examples of best practices already in place is particularly important to ensure the adaptability and flexibility of the organisational model to the different local healthcare settings and to underline that different solutions can be followed and applied in the same organisational model (e.g. ulcer medication clinic that can be organised as an *in house* or as an *outsourcing* service).

Expected output: Detailed list of the Key Performance Indicators (KPI).

### # Phase 5: refinement

*Aim* Development of the final version of the optimised common care pathway model, of related KPIs, and of instructions for its application in specific healthcare contexts.

After Phases 3 and 4, it is important to assess the eventual inclusion of additional KPIs and instructions that can be useful to implement the common organisational model. This should be done at HCP level, interacting with the multidisciplinary team, hospital management team, patients’ organisations, etc., in order to identify eventual KPIs that could be added, based on the single local framework and experience.

Once the common PCP model is finalised, a dissemination plan should be developed and followed by the stakeholders involved.

*Expected output* Inclusion of additional KPIs in the optimised common reference organisational model on the patients’ care pathway to be followed for the specific disease. Dissemination of the optimised common reference organisational model across the different stakeholders.

### # Phase 6: pilot phase (optional)

*Aim* To assess the applicability and adaptability of the optimised common patients’ care pathway in specific healthcare providers using related KPIs.

A pilot phase of a minimum 1-year period can be planned in order to assess how the optimised common PCP can be applied and adapted to the specific HCPs’s setting. This phase is particularly important to capture any possible barriers to the application of the organisational flow in a specific context and to identify further specific KPIs to be monitored for this purpose.

*Expected output* Application of the organisational model and collection of the KPIs. Identification of eventual barriers to the application of the organisational flow in specific contexts.

## Discussion

The need to develop a specific methodology for the implementation of an organisational model for PCP aligns with the mission of the ERNs to improve the care of RCD in Europe. In this framework, ERN ReCONNET developed the RarERN Path methodology that is aimed at creating an optimised common organisational model for PCP taking advantage of the unique setting of ERNs, represented by the large community of experts (researchers, clinicians, healthcare professionals, patients, health economists, hospital managers, etc.) involved in the Networks.

The RarERN Path methodology has already been applied in different diseases of the ERN ReCONNET thanks also to the collaboration with the Health Economics experts of the Institute of Management of the Scuola Superiore Sant’Anna. The first pilot disease in which the methodology was applied was Systemic Sclerosis, considering the high level of complexity of the disease and the coverage by the majority of the HCPs included in ERN ReCONNET. The methodology was particularly appreciated by the community of patients and clinicians that recognised the potential benefit of the application of this tool at different levels and its potential impact in the delivery of a better organisation of care to rCTDs patients.

One of the main innovative aspects of RarERN Path is represented by the active engagement of patients and patients’ representatives in the different phases of the process, which is important to ensure the patient-centricity of the approach and of the PCP itself. The collection and integration of patients’ voices and perspectives from large communities provides crucial added value, showing the high impact of a collaborative Network of patients and clinicians. Proof of this added value is the “ERN ReCONNET: Patients’ Stories” book series, an affiliated project of RarERN Path that collects all the patients’ stories in the different languages during Phase 2. The stories also represent a considerable contribution to raising awareness of the impact of the diseases on society as well as on the journey that patients with the disease face in their daily life. Besides patients, the methodology ensures the involvement of the main stakeholders involved in the provision of care of RCD and, for this reason, it is crucial that in the Consensus phase all the actors are included in the discussion.

Another important element to be considered is definitely the role that the optimised common organisational PCP model can play in the improvement of the application of CPGs in the different HCPs. The organisational PCP can in fact provide tangible support to the adoption of specific organisational procedures necessary to integrate the best practice indicated by the CPGs, resulting in better care for RCD patients. In addition, the application of the RarERN Path methodology can also provide useful results in order to increase the efficiency of the resources used for the diseases involved, as well as potentially more appropriate healthcare planning both at local and national level.

Furthermore, it is important to highlight that even though the RarERN Path methodology was developed in the framework of the ERN ReCONNET, the method was specifically designed to enable its application in different diseases contexts, thus, this procedure can be considered and applied in different ERNs, ensuring a shared and common transversal approach across all the Networks.

### The benefit of RarERN Path for European Reference Networks from the point of view of the European Commission

The ERN system is a new and unique organisational model of cross-border healthcare collaboration in the European Union (EU). The ultimate goal of the ERNs is to improve the diagnosis and treatment of patients suffering from rare or low prevalence and complex diseases. Several challenges should be overcome to reach this objective: the low number of cases of the diseases that may affect only a handful of patients in the EU and the lack of scientific knowledge of the diseases as well as their diagnosis and treatments. Situations such as these often result in delays in the diagnosis and non-appropriated treatments. There is a clear need for efficient, well-designed referral systems and patient pathways to allow patients to access the right providers, as well as the right techniques to benefit from the correct diagnosis, treatment and subsequent care.

Many of these challenges can be addressed by the ERNs, but this can only be possible through the full integration of the networks into national healthcare systems. National authorities, healthcare professionals, patients’ organisations and the European Commission are all working together towards this goal in order to ensure patient access to the ERN system while supporting the national healthcare system.

PCP represent a key multidisciplinary healthcare management tool in the ERN environment. These pathways are based on efficient healthcare planning and referral systems that would help better define the different appropriate tasks (or interventions) of the professionals involved in the patient care. Furthermore, they would improve how access to any of these interventions is structured. Costs and expected outcomes are principle elements which need to be considered throughout the entire chain of care and patient journey.

The recent statement of the ERN Board of Member States^1^ clearly states the need to advance in the development of effective patient pathways at national level and to link them to the current knowledge evidenced by the CPGs and the concrete organisational characteristics of each national healthcare system.

The RarERN Path methodology presented in this article represents an important step in that direction and the European Commission very much looks forward to the outcomes of this project, as it trusts it will be an important step forward in the effective implementation of the ERNs.

## Conclusions

The RarERN Path method intends to provide a feasible and pragmatic approach to implement and create a reference organisational model for PCP.

The application of RarERN Path to the different disease-specific and geographical contexts would help to ensure an improved, cost-effective and patient-centred equal care to RCD across Europe.

## Data Availability

Data sharing is not applicable to this article as no datasets were generated or analysed during the current study.

## Abbreviations

BoMS: ERN Board of Member States
CPGs: Clinical practice guidelines
ePAG: European Patients Advocacy Group
ERN ReCONNET: European Reference Networks for rare and complex connective tissue and musculoskeletal diseases
ERN: European Reference Networks
EU: European Union
HCP: Healthcare provider
KPI: Key performance indicators
PCP: Patients’ care pathways
RCD: Rare and complex diseases
rCTDs: Rare and complex connective tissue and musculoskeletal diseases
RDs: Rare diseases

## Footnotes

1 https://ec.europa.eu/health/sites/health/files/ern/docs/integration_healthcaresystems_en.pdf.
