# Correction to: RarERN Path: a methodology towards the optimisation of patients’ care pathways in rare and complex diseases developed within the European Reference Networks

**DOI:** 10.1186/s13023-021-01778-5

**Published:** 2021-03-22

**Authors:** Rosaria Talarico, Sara Cannizzo, Valentina Lorenzoni, Diana Marinello, Ilaria Palla, Salvatore Pirri, Simone Ticciati, Leopoldo Trieste, Isotta Triulzi, Enrique Terol, Anna Bucher, Giuseppe Turchetti

**Affiliations:** 1 Rheumatology Unit, Azienda Ospedaliero Universitaria Pisana, 56126 Pisa, Italy; 2 Institute of Management, Scuola Superiore Sant’Anna, Piazza Martiri Della Libertà, 33, 56127 Pisa, Italy; 3 DG Health and Food Safety, 1000 Brussels, Belgium

## Correction to: Orphanet J Rare Dis (2020) 15:347 https://doi.org/10.1186/s13023-020-01631-1

Following the publication of the original article [1] we were informed that the authors’ given and family names had unfortunately been interchanged.

The correct author names are shown here below:

Rosaria Talarico, Sara Cannizzo, Valentina Lorenzoni, Diana Marinello, Ilaria Palla, Salvatore Pirri, Simone Ticciati, Leopoldo Trieste, Isotta Triulzi, Enrique Terol, Anna Bucher and Giuseppe Turchetti.

The author names have been corrected in the author list of this Correction and updated in the original article.
